# Supplementary material for: Possible Role of Extracellular Vesicles in Hepatotoxicity of Acetaminophen
Source: Int J Mol Sci. 2022 Aug 9;23(16):8870. doi: 10.3390/ijms23168870 (PMC9408656; doi:10.3390/ijms23168870)
Supplement: Supplementary file 1 [file ijms-23-08870-s001.zip › Supplementary material - Experimental working scheme.pdf]

Experimental working scheme

Treatment of rats with a subtoxic dose of 500 mg/kg APAP,

euthanized 12h after administration

EVs collecting

perfusion of livers from control and APAP-treated rats (using Hank's balanced salt solution (HBSS) containing heparin; liver was perfused at 3 mL/min/g of liver)

EVs isolation

centrifugation at low speed (1000 x *g*, 5 min) and filtration through a 1.2µm filter

centrifugation of filtrate at 100 000 × *g*

sucrose gradient ultracentrifugation at 100,000 x g of the resuspended pellet (pellet from a single liver was resuspended in 1 mL PBS and layered onto a 27%/68% sucrose cushion)

EVs collected at the interface, were resuspended in 1 mL PBS, and submitted to fractionation by size exclusion chromatography

EVs sample preparation for MS

Gel-in-tube proteolytic digestion (EVs fractions were subjected to reduction and alkylation, incorporated into polyacrylamide gel in Eppendorf tubes, in-gel deglycosylated, followed by trypsin proteolytic digestion)

Detection of proteins by LC-MS/MS
