# Supplementary material for: Possible Role of Extracellular Vesicles in Hepatotoxicity of Acetaminophen
Source: Int J Mol Sci. 2022 Aug 9;23(16):8870. doi: 10.3390/ijms23168870 (PMC9408656; doi:10.3390/ijms23168870)
Supplement: Supplementary file 1 [file ijms-23-08870-s001.zip › Supplementary Tables 1-3.pdf]

Table S1. Proteins detected only in EVs of normal liver in comparison to EVs of acetaminophen treated liver.

Protein Name	Uniprot ID	Total Score	Peptides
UDP-glucuronosyltransferase	F1M7N8	14,15	7
Epoxide hydrolase 1	P07687	12,69	6
Long-chain-fatty-acid--CoA ligase 1	P18163	9,69	3
Serum albumin	P02770	9,63	4
Dehydrogenase/reductase (SDR family) member 7	Q6I7R1	9,61	4
Actin, cytoplasmic 2	P63259	9,51	5
Calnexin	P35565	9,17	5
NADPH-dependent 3-keto-steroid reductase Hsd3b5	P27364	8,66	4
UDP-glucuronosyltransferase 1-5	Q64638	9,41	5
Histone H4	P62804	7,09	3
NADH-cytochrome b5 reductase 3	P20070	6,96	3
L-gulonolactone oxidase	P10867	6,91	3
Ig gamma-2B chain C region	P20761	6,29	2
Cytochrome b5	P00173	6,23	3
Cytochrome P450 2B3	P13107	6,18	3
Cytochrome P450 2A2	P15149	5,9	2
Dimethylaniline monooxygenase [N-oxide-forming] 1	P36365	5,43	2
UDP-glucuronosyltransferase 2B15	P36511	7,13	3
Transitional endoplasmic reticulum ATPase	P46462	4,88	2
Serum paraoxonase/arylesterase 1	P55159	4,86	1
NADPH--cytochrome P450 reductase	P00388	4,59	2
Cytochrome P-450	Q06884	4,53	2
Cytochrome P450 2C6	P05178	5,19	2
ATP synthase subunit beta, mitochondrial	P10719	4,3	1
Cytochrome P450 2E1	P05182	4,17	2
UDP-glucuronosyltransferase 2B1	P09875	6	3
Fibronectin	P04937	3,71	2
Microsomal glutathione S-transferase 1	P08011	3,52	2
Dimethylaniline monooxygenase [N-oxide-forming] 3	Q9EQ76	3,43	1
Bile acyl-CoA synthetase	Q9ES38	3,32	1
Sterol-4-alpha-carboxylate 3-dehydrogenase, decarboxylating	Q5PPL3	3,22	1
Cytochrome P450 2D4	Q64680	14,73	8
Estradiol 17-beta-dehydrogenase 2	Q62730	2,74	1
Vesicle-trafficking protein SEC22b	Q4KM74	2,68	1
Cytochrome P450 4F1	P33274	2,5	1
Membrane-associated progesterone receptor component 1	P70580	2,48	1
60S ribosomal protein L24	P83732	2,48	1
40S ribosomal protein S25	P62853	2,48	1
Dolichyl-diphosphooligosaccharide--protein glycosyltransferase 48 kDa subunit	Q641Y0	2,42	1
60S ribosomal protein L13a	P35427	2,21	1
60S acidic ribosomal protein P0	P19945	2,21	1
Estradiol 17-beta-dehydrogenase 11	Q6AYS8	2,14	1
Cytochrome P450 2C23	P24470	2,21	1
Anionic trypsin-1	P00762	2,1	1
60S ribosomal protein L7a	P62425	2,02	1
Murinoglobulin-1	Q03626	2,9	1
Very-long-chain enoyl-CoA reductase	Q64232	2,01	1
UDP-glucuronosyltransferase 1-1	Q64550	9,41	5
60S ribosomal protein L29	P25886	2	1
60S ribosomal protein L4	P50878	2	1
Ig kappa chain C region, A allele	P01836	2	1
60S ribosomal protein L21	P20280	2	1
FERM, ARHGEF and pleckstrin domain-containing protein 1	F1LYQ8	2	1

60S acidic ribosomal protein P2	P02401	2	1
60S ribosomal protein L3	P21531	2	1
Transmembrane emp24 domain-containing protein 10	Q63584	2	1
Cytochrome b5 type B	P04166	2	1
Apolipoprotein E	P02650	2	1
40S ribosomal protein S19	P17074	1,8	1
Serine protease inhibitor A3L	P05544	1,71	1
D-beta-hydroxybutyrate dehydrogenase, mitochondrial	P29147	1,7	1
60S ribosomal protein L23a	P62752	1,7	1
40S ribosomal protein S30	P62864	1,7	1
Ceramide synthase 2	Q3T1K1	1,57	1
Cytochrome P450 1A2	P04799	1,52	1
Transmembrane protein 33	Q9Z142	1,52	1
40S ribosomal protein S28	P62859	1,52	1
60S ribosomal protein L17	P24049	1,33	1

Table S2. Proteins detected only in EVs of acetaminophen treated liver in comparison to EVs of normal liver.

Protein Name	Uniprot ID	Total Score	Peptides
Fibronectin	F1LST1	32,02	16
Sodium/potassium-transporting ATPase subunit alpha-1	P06685	25,53	13
Clathrin heavy chain 1	P11442	21,73	10
Actin, cytoplasmic 1	P60711	18,99	11
Tln1 protein	Q498D4	16,61	7
4F2 cell-surface antigen heavy chain	Q794F9	16,26	8
Integrin subunit alpha 2b	D3ZAC0	15,59	7
5'-nucleotidase	P21588	12,41	6
Annexin A1	P07150	12,4	6
Annexin A3	P14669	11,14	5
Metalloreductase STEAP4	Q4V8K1	10,78	5
Annexin A2	Q07936	10,56	5
Elongation factor 1-alpha 1	P62630	9,99	5
Guanine nucleotide-binding protein G(i) subunit alpha-2	P04897	8,79	4
Aminopeptidase N	P15684	8,71	4
Endothelial type gp91-phox	Q9ER28	8,55	4
Gene_Symbol=LOC100360950 GF20391-like isoform 1		8,1	8
Receptor-type tyrosine-protein phosphatase C	P04157	7,95	3
Ras-related protein Rab-10	P35281	7,7	4
MHC class Ia protein	O19446	7,67	4
Integrin beta 2	B2RYB8	7,64	4
Alpha-actinin-1	Q9Z1P2	7,52	4
Guanine nucleotide-binding protein G(I)/G(S)/G(T) subunit beta-2	P54313	5,84	3
Gene_Symbol=Ywhaz 14-3-3 protein zeta/delta	P63102	5,41	3
Annexin A5	P14668	5,1	2
Basigin	P26453	5,1	2
Plexin B2	D3ZQ57	5,04	2
Ras-related protein Rab-7a	P09527	4,99	2
Dipeptidase 1	P31430	4,86	2
ADP-ribosyl cyclase/cyclic ADP-ribose hydrolase 1	Q64244	4,7	3
Fibrinogen gamma chain	P02680	4,27	2
Monocyte differentiation antigen CD14	Q63691	4,17	2
Monocarboxylate transporter 1	P53987	4,17	2
Solute carrier family 22 member 7	Q5RLM2	4,15	2
Peroxiredoxin-1	Q63716	4,08	2
Solute carrier organic anion transporter family member 1B2	Q9QZX8	4	2
Solute carrier family 39 (Zinc transporter), member 14 (Predicted)	D3ZZM0	4	2
Solute carrier family 22 member 1	Q63089	4	2
40S ribosomal protein S15a	P62246	3,9	2
Catenin (Cadherin associated protein), delta 1 (Predicted), isoform	D3ZZZ9	3,7	2
40S ribosomal protein S9	P29314	3,68	1
Purine nucleoside phosphorylase	P85973	3,67	1
Arginase-1	P07824	3,62	1
Integrin alpha-1	P18614	3,58	2
Integrin beta-1	P49134	3,24	1
Bone marrow stromal antigen 2	Q811A2	3,22	1
Cell division control protein 42 homolog	Q8CFN2	3,22	2
ATP-binding cassette, sub-family A (ABC1), member 8a	D3ZCF8	3,16	1
Guanine deaminase	Q9WTT6	3,15	1
Gene_Symbol=Ehd3 EH domain-containing protein 3	Q8R491	3,1	1
Ras-related protein Rap-1b	Q62636	3	1
40S ribosomal protein S10	P63326	3	1

Heat shock protein HSP 90-beta	P34058	2,99	1
Tyrosine-protein kinase HCK	P50545	2,96	1
Sodium/potassium-transporting ATPase subunit beta-3	Q63377	2,96	1
Ninjurin-1	P70617	2,94	1
Sodium/potassium-transporting ATPase subunit beta-1	P07340	2,81	2
UDP-glucuronosyltransferase 2B37	P19488	2,57	1
Fc fragment of IgG receptor Ia	A0A0B4J2J0	2,46	1
MHC class I RT1.Ab heavy chain	Q31254	6,67	3
ATP synthase subunit beta, mitochondrial	P10719	2,37	1
GPI-anchored ceruloplasmin	Q9JL97	2,36	1
Catenin (Cadherin associated protein), alpha 1	Q5U302	2,31	1
40S ribosomal protein S26	P62856	2,29	1
Fibrinogen beta chain	P14480	2,28	1
Peptidyl-prolyl cis-trans isomerase A	P10111	2,27	1
Sulfate anion transporter 1	P45380	2,11	1
60S ribosomal protein L9	P17077	2,1	1
40S ribosomal protein SA	P38983	2,1	1
Ras-related protein Rab-1A	Q6NYB7	6,06	3
Tubulin alpha-4A chain	Q5XIF6	9,53	5
Phosphoglycerate kinase 1	P16617	2	1
Myristoylated alanine-rich C-kinase substrate	P30009	2	1
C-type lectin domain family 2, member G	A0A0G2JVV1	2	1
F-actin-capping protein subunit alpha-1	B2GUZ5	2	1
Betaine--homocysteine S-methyltransferase 1	O09171	2	1
C5a anaphylatoxin chemotactic receptor 1	P97520	2	1
Brain acid soluble protein 1	Q05175	2	1
Endoplasmin	Q66HD0	2	1
Aldehyde dehydrogenase 8 family, member A1	D3ZXY4	2	1
Platelet glycoprotein Ib beta chain	Q9JJM7	2	1
Carbonic anhydrase 14	A2IBE0	2	1
V-set and immunoglobulin domain-containing 4	F7FEU1	2	1
Gene_Symbol=Cd151 CD151 antigen	Q9QZA6	2	1
Thrombospondin 1	Q71SA3	1,78	1
Solute carrier family 2, facilitated glucose transporter member 2	P12336	1,72	1
Xanthine dehydrogenase/oxidase	P22985	1,7	1
CD44 antigen	P26051	1,7	1
Solute carrier organic anion transporter family member 2A1	Q00910	1,65	1
Ras-related protein Rab-1B	P10536	5,58	3
MHC class II antigen	Q6T4R6	1,52	1
Ras-related protein Rab-11B	O35509	1,52	1
ADP-ribosylation factor 6	P62332	1,52	1
40S ribosomal protein S24	P62850	1,52	1
Guanine nucleotide-binding protein G(I)/G(S)/G(T) subunit beta-1	P54311	5,24	3
40S ribosomal protein S7	P62083	1,4	1
Eukaryotic initiation factor 4A-II	Q5RKI1	1,4	1
Sushi domain containing 2 (Predicted), isoform CRA_a	D3ZEV8	1,4	1
Solute carrier family 2 member 9	D4A237	1,4	1
Histone H2A	D3ZVK7	5,34	2
Integrin alpha L	Q3T1L6	1,3	1

Table S3. Proteins detected in EVs of both, acetaminophen treated liver (Treated EV) and normal liver (NLEV).

Protein Name	UNIPROT	Total score (Treated EV)	Total score (NLEV)	Peptides (Treated EV)	Peptides (NLEV)
Major vault protein	Q62667	31,65	13,43	13	6
Hemoglobin subunit beta-2	P11517	17,75	14,25	10	7
Tubulin beta-5 chain	P69897	13,68	4	7	2
Hemoglobin subunit alpha-1/2	P01946	12,78	11,22	6	6
Cytochrome P450 2D26	P10634	12,72	33,98	5	18
Integrin beta-3	Q8R2H2	11,13	2,02	5	1
Tubulin alpha-1C chain	Q6AYZ1	10,47	3,52	5	2
Band 3 anion transport protein	P23562	10,23	9,4	5	3
Solute carrier organic anion transporter family member 1A4	O35913	9,79	2	5	1
Annexin A6	P48037	9,88	3,51	3	1
Inter alpha-trypsin inhibitor, heavy chain 4	Q5EBC0	8,61	4	4	2
Glyceraldehyde-3-phosphate dehydrogenase	P04797	8,44	4,62	3	2
Filamin A	C0JPT7	7,15	1,59	3	1
Retinol dehydrogenase 7	P55006	7,1	15,55	2	8
40S ribosomal protein S13	P62278	7,01	1,41	3	0
Alpha-1-macroglobulin	Q63041	6,3	57,95	2	25
Myosin-9	Q62812	5,85	2,8	2	1
Fibrinogen alpha chain	P06399	5,63	2	2	1
40S ribosomal protein S4, X isoform	P62703	5,6	1,96	1	0
Cytochrome P450 2D1	P10633	6,38	28,33	2	14
40S ribosomal protein S6	P62755	4,85	2,84	2	1
Ferritin light chain 1	P02793	4,4	9,52	2	5
40S ribosomal protein S16	P62250	4,25	4,52	2	2
60S ribosomal protein L6	P21533	4,08	4,06	2	2
Carbamoyl-phosphate synthase [ammonia], mitochondrial	P07756	4,01	1,47	1	0
Hemoglobin subunit beta-1	P02091	16,96	14,1	11	7
60S ribosomal protein L7	P05426	2,68	2,52	1	1
60S ribosomal protein L18	P12001	2,48	6,41	1	4
Cytochrome P450 2C11	P08683	2,44	15,83	1	6
60S ribosomal protein L15	P61314	2,44	3,78	1	2
Vitronectin	Q3KR94	2,25	2,44	1	1
Cytochrome P450 4A2	P20816	2,2	9,84	1	4
Corticosteroid 11-beta-dehydrogenase isozyme 1	P16232	2,11	5,16	1	3
Retinol dehydrogenase 16	P50170	6,99	10,96	2	6
60S ribosomal protein L26	P12749	2,01	2,35	1	1
60S ribosomal protein L10	Q6PDV7	2,01	2,87	1	1
40S ribosomal protein S3	P62909	2	1,57	0	1
40S ribosomal protein S8	P62243	2	2,09	1	1
60S ribosomal protein L8	P62919	2	2,44	1	1
Ferritin heavy chain	P19132	2	5,65	1	2
60S ribosomal protein L14	Q63507	2	2	1	1
Aldehyde dehydrogenase family 3 member A2	P30839	2	12,43	1	6
Receptor expression-enhancing protein 6	Q5XI60	2	4	1	2
40S ribosomal protein S14	P13471	2	2	1	1
60S ribosomal protein L23	P62832	1,78	2	1	1
60S ribosomal protein L27a	P18445	1,7	1,63	1	1
