# Supplementary figures and images for: Possible Role of Extracellular Vesicles in Hepatotoxicity of Acetaminophen
Source: Int J Mol Sci. 2022 Aug 9;23(16):8870. doi: 10.3390/ijms23168870 (PMC9408656; doi:10.3390/ijms23168870)

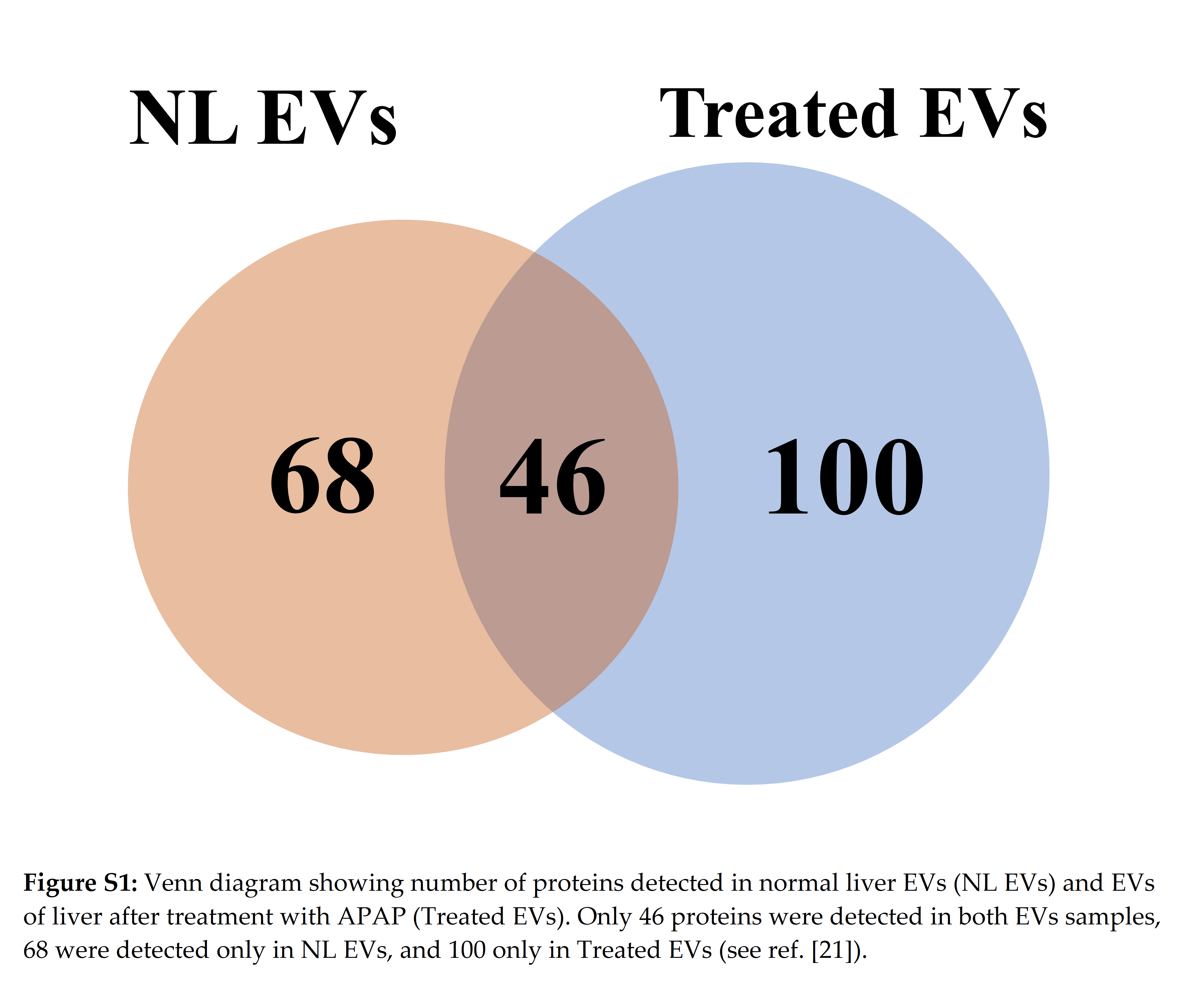

Supplement: Supplementary file 1 [file ijms-23-08870-s001.zip › Figure S1.tif]
